# Biochemical and Hematological Relationship with the Evaluation of Autonomic Dysfunction by Heart Rate Recovery in Patients with Asthma and Type 2 Diabetes

**DOI:** 10.3390/diagnostics11122187

**Published:** 2021-11-24

**Authors:** O. Gamaliel Aztatzi-Aguilar, Claudia Vargas-Domínguez, Yazmin Debray-Garcia, Manolo S. Ortega-Romero, Paloma Almeda-Valdés, Carlos A. Aguilar-Salinas, M. Augusta Naranjo-Meneses, D. Abril Mena-Orozco, César E. Lam-Chung, Ivette Cruz-Bautista, M. Patricia Sierra-Vargas

**Affiliations:** 1 Departamento de Investigación en Toxicología y Medicina Ambiental, Instituto de Enfermedades Respiratorias Ismael Cosío Villegas, Ciudad de México 14080, Mexico; gammaztatzi@gmail.com (O.G.A.-A.); clauv8@gmail.com (C.V.-D.); yazmindebrayg@gmail.com (Y.D.-G.); rom_0@hotmail.com (M.S.O.-R.); 2 Centro de Investigación y de Estudios Avanzados, Instituto Politécnico Nacional, Ciudad de México 07360, Mexico; 3 Unidad de Investigación en Enfermedades Metabólicas, Instituto Nacional de Ciencias Médicas y Nutrición Salvador Zubirán, Ciudad de México 14080, Mexico; paloma.almedav@incmnsz.mx (P.A.-V.); caguilarsalinas@yahoo.com (C.A.A.-S.); maguname@yahoo.com (M.A.N.-M.); abril.mo@gmail.com (D.A.M.-O.); cesar.lamc@incmnsz.mx (C.E.L.-C.); ivette.cb27@gmail.com (I.C.-B.); 4 Subdirección de Investigación Clínica, Instituto de Enfermedades Respiratorias Ismael Cosío Villegas, Ciudad de México 14080, Mexico; 5 Facultad Mexicana de Medicina, Universidad La Salle, Ciudad de México 14000, Mexico

**Keywords:** biochemical evaluation, hematological evaluation, autonomic dysfunction, heart rate recovery, 6-min walking test

## Abstract

There are several methods to assess the function of the autonomic nervous system. Among them, heart rate recovery (HRR) is an accepted, easy, low-cost technique. Different pathological conditions have been related to the development of autonomic dysfunction. Our study aimed to evaluate the relationship between HRR and HRR-derived parameters in ambulatory patients with asthma or type 2 diabetes followed at the National Institutes of Health in Mexico City. A total of 78 participants, 50 women and, 28 men were enrolled; anthropometric, respiratory evaluations, and fasting blood samples were taken before participants performed a 6-min walking test (6MWT). Abnormal HRR was defined as a drop of ≤8 and ≤11 beats/min at 1 or 2 min and correlated negatively with basal oxygen saturation at 1 min. Heart rate at 1 min, correlated negatively with final oxygen saturation (*p* < 0.01). Statistically significant negative correlations were also observed between red cell count and white blood cell count and HOMA-IR with a *p* < 0.01. Since discrete hematological but significant changes correlated with HRR and HRR-derived parameters, we consider that these measures are helpful in clinical settings to identify subclinical autonomic dysfunction that permits us to prevent or anticipate chronic and fatal clinical outcomes.

## 1. Introduction

The autonomic nervous system (ANS) is in charge of a series of reflex reactions. The maintenance of a stable internal environment depends on the balance between the sympathetic and parasympathetic systems. Changes in the activity of ANS can occur temporarily, but the majority of chronic diseases are associated with an important dysregulation of ANS. Asthma, diabetes, and obesity, common chronic diseases, are associated with each other as comorbidity, but also linked to meta-inflammation where a myriad of manifestations of autonomic alterations has been identified [1,2]. Those diseases share common risk factors also related to lifestyle and the increase of adiposity. For example, the study of Popa et al. showed that 61.7% of the population studied consumed fast food at least three times a week, increasing the risk for obesity 1.9 times, moreover 52.3% performed less than 150 min/week of physical exercise increasing the risk for such a condition 1.8 times [3]. Undoubtedly, an increase in adipose tissue leads to clinical obesity. This increment frequently results in the secretion of adipokines, which in turn causes low-grade systemic inflammation. However, there is a connection between inflammation and ANS that regulates the pro and anti-inflammatory response in a dependent manner. The reactivation of chronic inflammatory illnesses like asthma is related to ANS activation. Likewise, overproduction of interleukin 6 by endothelial cells is also linked to ANS [4]. To evaluate the functionality of ANS several tests are available. Most of them require specialized equipment and trained staff to perform the evaluation. Parasympathetic responses decrease with increasing body mass index (BMI) [5]. Recently, a considerable amount of attention has been paid to hematological parameters as an innovative tool to evaluate a variety of ASN alterations related to an acute coronary syndrome in the hospital setting. [6]. The rate of recovery of heart rate (HR) within the first minute after cessation of exercise, termed heart rate recovery (HRR), is a simple, accepted measure of ANS dysfunction, reflecting parasympathetic reactivation. The 6-min walking test (6MWT) is a standard, widely available, clinical test of cardiopulmonary reserve of patients with heart failure and pulmonary diseases like pulmonary hypertension, chronic obstructive pulmonary disease (COPD), and idiopathic pulmonary fibrosis (IPF). The 6MWT is a submaximal exercise test and a simpler alternative to the cardiopulmonary exercise test [7,8].

Different abnormal HRR (aHRR) cutoffs have been utilized in the past. For example, a cutoff of ≤42 beats/min after 2 min of recovery predicted cardiovascular death. A cutoff of ≤22 beats/min during the first 2 min after exercise anticipated the presence of coronary artery disease and death in males. Moreover, a decline of ≤42 beats/min after 2 min is associated with impaired fasting plasma glucose and an abnormal triglycerides/HDL cholesterol ratio (highest quartile) in healthy adults. Clinical deterioration in idiopathic pulmonary arterial hypertension is associated with less than 16 beats/min at the first minute. Abnormal HRR of 13 and 22 beats/min at the first and second minute, respectively, are good predictors of mortality in idiopathic pulmonary fibrosis. In chronic obstructive pulmonary disease (COPD), a cutoff of ≤12 beats/min in the first minute is related to pulmonary function, oxygen saturation, and disease severity [9].

HRR is a predictive biomarker of clinical worsening, hospitalization, and survival of different diseases such as connective tissue disease-associated pulmonary hypertension, idiopathic pulmonary fibrosis, poorly controlled severe asthma, cystic fibrosis, chronic obstructive pulmonary disease (COPD), bronchiectasis, heart failure, and diabetes [10,11,12]. HRR correlates with nutritional state and biochemical parameters. Some studies stated that HRR values at first (HRR1) and second (HRR2) minute post-exercise did not differ substantially from each other and are a significant risk factor for death from any cause, cardiovascular death, and even non-cardiovascular death [13,14].

Acute coronary syndrome has been associated with changes in blood cell count [14] and proposed as biomarkers of prognosis in those patients but there is not enough recent evidence regarding the relationship between cellular and biochemical measurements commonly used in clinical settings with HRR. The goal of this study was to evaluate the relationship between HRR and anthropometry, body composition, hematological and blood chemistry parameters, and whether these have possible use as autonomic dysfunction biomarkers.

## 2. Materials and Methods

We undertook this study at the Instituto Nacional de Enfermedades Respiratorias Ismael Cosío Villegas (INER) in Mexico City at 2240 m above sea level and treating mainly uninsured patients. The institutional Ethics Committee granted ethical approval, code C22-14, and all the participants signed an informed consent form. Sensitive information was removed from the collected data to preserve the anonymity of the participants. We conducted the study under the ethical principles of the Declaration of Helsinki.

We conducted a cross-sectional study of ambulatory patients with controlled asthma from the Asthma Clinic of the INER and patients with type 2 diabetes (T2D) recruited from the nearby Instituto Nacional de Ciencias Médicas y Nutrición Salvador Zubirán (INCMNSZ). Males and females aged 18 to 65 years, free of major diabetes-related chronic complications, and displaying HbA1c levels < 12%, and healthy volunteers, were invited to participate. Patients with a previous diagnosis of a cardiac congenital condition, autoimmune disease (including type 1 diabetes), oncologic disease, total cholesterol > 200 mg/dL, triglycerides > 300 mg/dL, thyroid disease, a body mass index less than 20 or more than 40 Kg/m^2^, individuals who had smoked at least 100 cigarettes and who smoked at the time of the study, treatment with beta-blockers and patients with an acute respiratory infection or asthma exacerbation within the previous 6 weeks at the time of the study were excluded. We did not analyze data from patients unable to perform the 6MWT.

All participants were asked to stop short- and long-acting inhaled beta2 agonists as well as anticholinergic drugs such as ipratropium bromide 12 h before the 6-min walking test (6MWT). If they could not stop their medication, the 6MWT test was not performed. Body composition (BIA) was estimated with a bioimpedance analyzer (Seca^®^ mBCA-514 analyzer, Hamburg, Germany). Anthropometric measurements including neck and waist circumference, complete blood count (CBC), and blood chemistry (BC) were obtained. Respiratory function was evaluated with forced spirometry (NDD Easy On-PC, ndd Medical Technologies, Inc., Massachusetts, USA) following the ATS/ERS 2005 guidelines; fractional exhaled nitric oxide (FeNO) and exhaled carbon monoxide measurements were also performed. Exhaled carbon monoxide was measured with a Smokerlyzer^®^ (Bedfont Scientific Ltd., Harrietsham, UK); FeNO measurements were conducted at a constant flow of 50 mL·s^−1^ in line with the ATS/European Respiratory Society (ERS) recommendations using a portable handheld analyzer (NObreath^®^ analyzer; Bedfont Scientific Ltd., Harrietsham, UK). The 6MWT was performed indoors, along a flat, straight, 30 m walking course, according to the guidelines of ATS/ERS 2014 [15]. HRR was calculated as the difference between peak heart rate and heart rate 1, and 2 min following test cessation; relative HRR (rHRR) was obtained with the following formula:

(1) $$\left(\frac{HR_{f}-HR_{x}}{HR_{f}}\right)*100$$

where *HR_f_* is final heart rate and *HR_x_* is heart rate at rest.

Arterial blood pressure and blood samples were obtained before the 6MWT and the lung function tests; all tests were carried out during fasting at the same hour of the day to avoid circadian variations in the autonomic nervous system activity. Arterial blood pressure was measured after a 5-min rest in a seated position by auscultation of the brachial artery with a stethoscope (3M™ Littmann^®^ Classic III™, Two Harbors, MN, USA) and an aneroid sphygmomanometer (767 Mobile Aneroid Welch Allyn, Inc., Skaneateles Falls, NY, USA) to detect the appearance and disappearance of the Korotkoff sounds and performed by the same device and same pneumologist specialized in respiratory tests to minimize inter-device and interobserver variability. Blood pressure was categorized according to the American Heart Association criteria [16]. The laboratory analyses were performed at the institutional laboratory. The BC analysis included the following parameters: albumin, triglycerides, total cholesterol, high-density lipoprotein (HDL), low-density lipoprotein (LDL), no-HDL, phospholipids, apolipoprotein A (Apo-A), apolipoprotein B (Apo-B), glucose, glycosylated hemoglobin (HbA1c), Insulin, C-reactive protein (CRP), urea, blood urea nitrogen (BUN), uric acid, and creatinine. From the BC analysis, we obtained the following indices: homeostatic model insulin resistance (HOMA-IR); Castelli risk index I (total cholesterol/high-density lipoprotein) and Castelli risk index II (low-density lipoprotein/high-density lipoprotein); atherogenic coefficient (AC; (TC-HDL)/HDL); Apo index; and atherogenic index of plasma (AIP; Log10 (TG/HDLc)). To evaluate the relationship between HRR and the measured parameters, the patients were reclassified into several categories as follows: sex, BMI, presence or absence of metabolic syndrome according to the National Cholesterol Education Program’s Adult Treatment Panel III/NCEP-ATPIII, and diagnosis of type 2 diabetes mellitus. Insulin resistance (IR) was determined through homeostatic model assessment (HOMA-IR) using a calculator (https://amhigo.com/mi-diagnostico/calculadoras/indice-de-resistencia-a-la-insulina-homa-ir, accessed on 19 October 2019) with cutoffs for the Mexican population. We also obtained values for waist-to-height-ratio (WHtR) and neck circumference. Finally, based on the atherogenic indices, the groups were classified as low or high risk.

### Statistical Analysis

The baseline characteristics of the studied population were stratified as follows: abnormal HRR at minutes 1 (aHRR1) and 2 (aHRR2) with cutoff values set at ≤8 and ≤11 bpm, respectively, based on the first quartile of all the data; presence or absence of asthma; type 2 diabetes and metabolic syndrome (MetS); BMI classification; WHtR; and high or low atherogenic risk. 

All data are expressed as the median followed by the interquartile range 25–75 (IQR). Correlations between variables were performed with a Spearman’s test. Comparisons between more than two groups were made using a non-parametric Kruskal–Wallis test followed by a post-hoc Dunn’s test. A Wilcoxon rank-sum test was used to compare two groups. A chi-square test was used to compare frequencies between two categorical variables. All statistical analyses were conducted in STATA 13 (StataCorp. 2013. Stata Statistical Software: Release 13. College Station, TX, USA: StataCorp LP). Graphs were created using GraphPad Prism version 6.00 for Windows (GraphPad Software, La Jolla, CA, USA).

## 3. Results

### 3.1. Descriptive Analysis

A total of 78 participants were enrolled. The general characteristics of the population are summarized in Table 1. Most of the participants were women (64.1%) with a median age of 45 years. Approximately 38.1% of the participants were overweight and 37.1% were obese, 39.7 % had MetS, and 24.4% were diabetic. Based on the HOMA index, 24.6% of the participants were suspected of having IR and 36.2% fulfilled the criteria for IR. Moreover, 83.1% of the population studied was considered overweight or obese based on WHtR; this percentage falls to 70.1% and 58.9% if we consider neck and waist circumference, respectively. Phase angle is a BIA measurement that results from the reactance/resistance ratio, which relates to a ratio of fat-free mass (resistance) and body cell mass (reactance) [17]. Phase angle is a clinical tool used to identify nutritional risk and ageing, and a predictor of illness progression [18]. There are no international reference values for phase angle; however, a 5.4–5.7° range has been suggested for healthy adults and altered measurements could be associated with inflammatory markers in people with obesity and type 2 diabetes like C reactive protein [18]. Based on a cutoff of 5.4°, our results showed that 48.7% of the participants could be classified as malnourished. In addition, 41.4% had a medical diagnosis of asthma. According to the blood pressure measurements, 31.5% and 28.8% fell into the pre-hypertension and hypertension categories, respectively.

The atherogenic indices calculated related the studied population with a high risk for cardiovascular disease (CVD) Table 1.

Respiratory-related measurements and bioelectrical impedance, blood cell counts, and blood chemistry parameters are shown in Table 2. Most of the parameters were within the normal range. Impairment of the autonomic nervous system, evidenced by HRR, is present in the different groups studied. Despite the high frequency of CVD risk and metabolic condition in our patients, the proportions of aHRR1 and aHRR2 were similar. However, the chi-square analyses (data not shown) suggest that aHRR1 and aHRR2 do not have a relationship with sex, the diagnosis of diabetes, asthma, MetS, or nutritional state.

**Figure 1:**
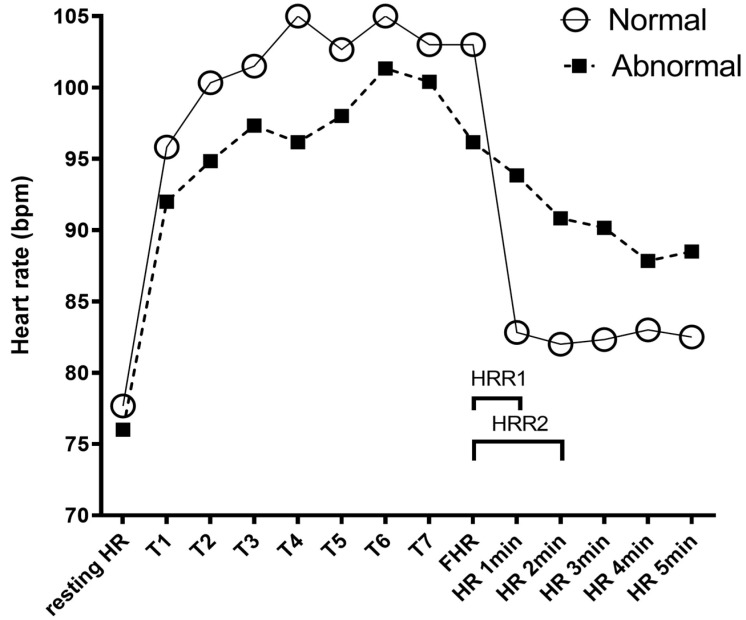
Representative graph of the heart rate (HR) evaluation during the 6-min walking test (6MWT). Resting-HR and oxygen saturation (SpO2) were recorded before the 6MWT with a pulse oximeter. After each turn (T), HR and SpO2 were recorded. The open circles show the HR of healthy patients throughout the 6MWT. The black squares show the HR of patients with abnormal heart rate recovery (HRR). At the end of the 6MWT, we recorded the final heart rate (FHR) and then recorded the HR each minute during the 5 following minutes. Heart rate recovery at the first minute (HRR1) was defined as FHR minus HR1min and HRR2 was calculated in the same way. To establish categorical abnormal HRR1 (aHRR) and HRR2 (aHRR), the first quartile of all the data, ≤8, and ≤11 bpm was used as cutoff point for worsening, respectively.

Relationship between HR, HRR, and rHRR from the 6MWT (indicators of a dysautonomic state) and general health parameters, can be seen in a representative graph in Figure 1.

### 3.2. Correlation Analysis

We included basal, final oxygen pulse saturation (SpO2b and SpO2f, respectively) and FeNO from the respiratory-related measurements. A statistically significant positive correlation between SpO2b and HRR1 (rho 0.309) and rHRR1 (rho 0.289) was observed (Table 3).

Our data showed a statistically significant difference in SpO2b between the HRR1 group (95%) and the normal aHRR1 group (93%). Moreover, SpO2f correlated negatively with HR1. However, SpO2f was lower in the aHRR1 group compared to the group with normal HRR1. There was also a difference in FeNO concentration between the compared HRR2 and aHRR2 groups (Table 3).

Several parameters of body composition showed statistically significant differences. We also observed that skeletal muscle mass (SMM) negatively correlated with rHRR1, but it displayed a trend to increase with aHRR1 and aHRR2 (Table 4). Phase angle (PA) correlated negatively with HRR1 (rho −0.23, *p* = 0.047), similarly, PA correlated negatively with rHRR1 (rho −0.26, *p* = 0.02) and rHRR2 (rho −0.27, *p* = 0.015) (Table 4). 

Intracellular water showed a negative correlation with rHRR1 (rho −0.227, *p* = 0.045), which was statistically significant. A statistically significant increase in intracellular water was observed in the aHRR1 group, 20.3 L (16.2–26), compared to those with normal HRR1, 17.05 L (15.1–20.3); *p* = 0.038, Table 4. 

Hydration percentage showed a statistically significant positive correlation with HRR1 (rho 0.234, *p* = 0.039), rHRR1 (rho 0.255, *p* = 0.024), and rHRR2 (rho 0.23, *p* = 0.042). Hydration was significantly lower in aHRR1 versus the normal HRR1 group (Table 4).

From the blood cell count (Table 2), we considered the total count of white blood cells that showed a statistically significant increase in aHRR1 subjects versus the HRR1 group, except for eosinophils and basophils, which did not show any statistical differences (data not shown). Leucocytes and neutrophils displayed a significant negative correlation with HRR1, HRR2, rHRR1 and rHRR2; these correlations were only observed in HRR1 and rHRR1 for lymphocytes and monocytes (Table 5).

Erythrocytes, hemoglobin, and hematocrit were included as red blood cell parameters (Table 2); all of them showed a statistically significant increase in aHRR2 group and a negative correlation with HRR and rHRR (Table 5) that was statistically significant only for hematocrit.

The blood chemistry parameters used in the present study are shown in Table 2. Blood glucose metabolism-related parameters, such as fasting glucose and HbA1c, did not correlate with HR, HRR, or rHRR. However, insulin and HOMA-IR had a positive and statistically significant correlation with HR1 (*p* < 0.01) and HR2 (*p* < 0.05). By contrast, HOMA correlated negatively with HRR1 (*p* < 0.05), rHRR1 correlated negatively with insulin (*p* < 0.05) and HOMA-IR (*p* < 0.05). We can observe an increase in HOMA-IR in aHRR1 subjects, with a marginal *p*-value (*p* = 0.052) (Table 6). 

As we mentioned earlier, there was a high prevalence of cardiovascular risk among the participants. rHRR2 showed a statistically significant correlation with Castelli-I (*p* < 0.05) but, this correlation was marginal with AC (*p* = 0.05), HR1 and HR2 displayed a positive correlation with some dyslipidemia parameters as can be seen in Table 6.

**Figure 2:**
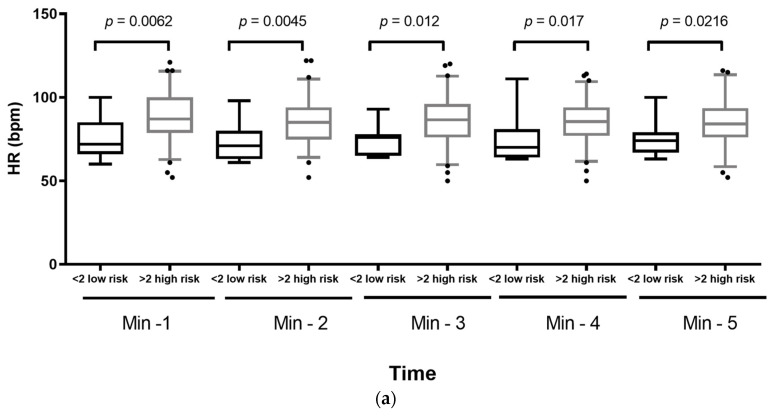

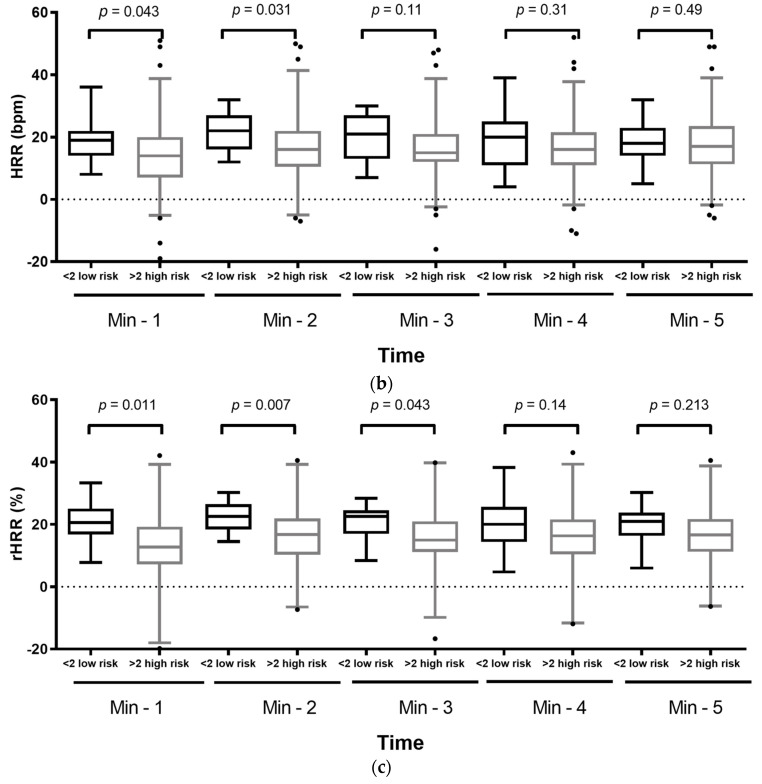
(**a**) A high atherogenic coefficient affects heart rate (HR), (**b**) heart rate recovery (HRR), and (**c**) relative heart rate recovery (rHRR). Boxplot graphs comparing low-risk < 2 atherogenic coefficient and high-risk > 2 atherogenic coefficient at each minute after the 6-min walking test. A Wilcoxon rank–sum test was used to compare the two groups; *p*-values are shown above each comparison.

Given that high atherogenic risk was observed among the participants, we grouped the subjects into low and high risk according to the AC and compared HR, HRR, and rHRR during the 5 monitored minutes. We observed that patients with high AC, established by a cutoff point of 2, showed a statistically significant increase in HR during the 5 min of rest after the 6MWT (Figure 2a). However, patients with high AC had significantly lower HRR1 and HRR2 compared with subjects with low AC (Figure 2b). When HRR was transformed to rHRR, we observed that, in addition to low rHRR1 and rHRR2 in the high-risk AC group, a statistically significant difference remained until rHRR3 (Figure 2c). The AC showed that patients with high no-HDL, which includes chylomicrons, VLDL, IDL, and LDL, increased the HR at each minute after the 6MWT. The bpm lost was lower in HRR as was the percentage of rHRR in the subjects with high-risk AC.

## 4. Discussion

The 6MWT is a clinically validated submaximal exercise test [8], similar to activities undertaken in everyday life with relevant association with patients’ symptoms, quality of life, all-cause mortality, hospital readmission, and the combined endpoint of death or read-mission in patients with heart failure [19]. Post-exercise HR and HRR evaluate vagal nerve integrity and measure cardiac autonomic activity.

BIA is a non-invasive assessment of tissues, based on electrical impedance, and affected by changes in health status (e.g., nutritional state, swelling, infections, and disease). Our study proposes a relationship between BIA measurements and the HR recovery after exercise, implying a dysautonomic state of the patients. Phase angle, intracellular water, and hydration percentage showed statistical differences related to dysautonomia as determined by a slow HRR. At present, there are different indices to establish nutritional condition (e.g., BMI, neck and waist circumference, waist-to-height ratio); however, BIA provides additional information regarding the nutritional state than other parameters. Phase angle showed a significant correlation with HR, HRR, and rHRR whereas other indicators of a nutritional condition such as BMI, WHtr, NC and WC, did not show any statistical association. 

The SMM parameter showed marginal and discrete statistical differences. This result suggests an increase in muscle mass, possibly cardiac muscle mass. Bioimpedance cannot distinguish between skeletal and visceral muscle mass. Disturbances of the autonomic nervous system are present in heart hypertrophy of different etiologies, such as hypertension or atherogenic risk, and heart failure to compensate for the cardiac output [20].

Studies report that the immune response can contribute to autonomic nervous system dysfunction through immune signaling molecules like cytokines, affecting heart rate variability [21], which correlates with HRR [22] through the depression of action potential [23]. The HRR results presented in this study suggest the presence of integrated immune-neuroendocrinal interactions, where white blood cell counts contribute to aHRR values. HRR depends on a physiological regulation mechanism. The sympathetic nervous system regulates the immunological cells, immune organs, and the responses of acute phase reactions [24]. The observed white blood cell count values suggest a relationship between circulatory inflammatory cells and changes in HRR1, rHRR1, and aHRR1. Even though the cell counts found in both HRR and aHRR subjects were within the normal reference limits, the differences between groups had statistical significance. A cross-sectional study displayed similar results; there was an increase in white blood cell count and PCR in aHRR1 subjects. This phenomenon was independent of disease status, blood pressure, blood lipids, body size, smoking, and fasting blood glucose [20].

Hematological parameters are related to oxygen transport and iron deficiency. Total erythrocytes, hemoglobin, and hematocrit showed a correlation and discrete changes related to HR recovery.

Insulin resistance syndrome contributes to HRR in adolescents, adults, and elderly men [25,26]. It correlated with malnutrition and a high frequency of metabolic syndrome as well. The increase in serum insulin is related to chronic inflammation, and both stimuli contribute to the overstimulation of the sympathetic nervous system. Additionally, several prospective studies indicate that impaired glucose tolerance at baseline is an independent predictor of cardiovascular disease, even among the nondiabetic population. Moreover, metainflammation is related to the development of atherosclerosis [21,27]. Our results are in accordance with the prevalence of dysautonomia in patients with CVD risk, which was observed in many of the patients in HRR during the first minute of rest after the maximal and submaximal exercise test [21]. Furthermore, we observed that this effect persisted within the first three minutes after the 6MWT suggesting that even when comorbidities such as MetS, diabetes, and asthma are under control, patients have a high cardiovascular risk that increases susceptibility to atherogenic processes. We observed that HR at 1 and 2 min after the 6MWT had a better correlation against the calculated HRR and rHRR. Therefore, HR should be considered together with HRR parameters.

In summary, this study describes respiratory-related parameters, body composition, and cellular and biochemical blood changes in a population with asthma, type 2 diabetes and obesity, under medical control. These medical conditions lower life expectancy due to a higher risk of developing fatal cardiovascular events. Furthermore, overweight and obesity per se are also significant risks for CVD and have a negative impact on fitness performance independent of age [21]. The 6MWT test should be included in the battery test in hospitalized and ambulatory patients as part of the evaluation of illness progression and treatment response.

The time difference in heart rate recovery after cessation of exercise is a biomarker of the outcome. Van de Vegte, et al. [22] reported an association of change in heart rate between 10 s and 1 min after exercise cessation and found that 10 s is a better predictor of mortality for all-cause and coronary artery disease mortality. The evaluation at 1 or 2 min after exercise cessation is used in clinical and sports assessment. Lamberts et al. [23] reported that HRR helps to monitor changes in endurance performance and contributes to a more accurate prescription of training load in well-trained and elite cyclists. High HRR in athletes is related to cardiovascular fitness; however, research in athletes is limited; moreover, the methodologies to evaluate HRR used in these studies are varied [28]. On the other hand, clinical HRR has been used as a predicted biomarker for worsening and deadly cardiorespiratory and metabolic diseases. Few studies aim to explain the cellular and biochemical parameters involved in nervous dysautonomia.

Our study suggests that HR, HRR, and rHRR are related to biochemical, cellular, and physiological responses. Patients with dysautonomia showed lower oxygen saturation that harms the microvasculature because of oxygen restriction. These physiological changes contribute to the disruption of the autonomous nervous system. They also promote subclinical immunological and hematological modifications to compensate for a hypoxic environment. Immune cells promote inflammation that contributes to insulin resistance syndrome concomitant with dysregulation of the hydric state, expressed by water body composition. Inflammation and insulin resistance favor autonomic nervous dysregulation and the integrity of the vagal nerve.

Heart rate variability (HRV) is another clinical tool used to evaluate autonomic nervous dysregulation. One methodological advantage of HRR using the 6MWT over HRV is the procedure and the cost of the equipment, which is more expensive for HRV compared to the 6MWT; however, HRV statistical or domain parameters are more refined than those of resting HR or the estimation of HRR. Nevertheless, the use of HRR methodology results in a low-cost test that is easy to perform and quickly interpreted by health or sports staff. Even though both HRV and HRR are accepted as non-invasive measurements of autonomic dysfunction, and useful biomarkers for worsening and death prognosis, there is still a debate about the lack of correlation between HRR and HRV, influenced by independent aspects of cardiac function (respiratory frequency, temperature, noise) [29]. These two types of measurement provide self-sufficient and complementary information on cardiac parasympathetic function. Further studies are needed to establish a better correlation and determine how biochemical and hematological biometry explain or contribute to autonomic dysfunction. 

Limitations of the present study are the low number of patients recruited and the poor relation of the biochemical and hematological parameters to the extended reported outcomes related to HRR, such as survival and clinical worsening. However, a relevant contribution of the present study is the relation of HRR and biochemical, cellular, and physiological parameters that can explain autonomic dysfunction and the increase of atherogenic risk to this condition. 

## 5. Conclusions

Evaluation of HR, HRR, and rHRR after a 6MWT, biomarkers of autonomic dysfunction, are simple clinical tools, responding to discrete changes in respiratory-related measures, body composition, blood cell count, and blood chemistry. Those biomarkers may identify abnormalities in apparently healthy patients that could prove clinically relevant for prognosis and treatment selection and proper follow-up.

## Figures and Tables

**Table 1:** General characteristics of the population recruited by National Institutes of Health, Mexico City.

**Variable**	**Total (*n* = 78)**		
	**Median (IQR)**		**Min-Max**
**Age (Years)**	45 (34–53)		25–67
**Anthropometry**			
Height (m)	1.59 (1.53–1.67)		1.44–1.78
Weight (kg)	71.55 (61.8–82.3)		47–106.8
Waist Circumference (cm)	92 (86–102)		59–117.5
Neck Circumference (cm)	35.75 (32.5–39)		27–49
WHtR	0.57 (0.53–0.65)		0.39–0.79
BMI (kg/m^2^)	27.8 (25–30.8)		20.3–38.5
**Variable**	**N (%)**	**Variable**	**N (%)**
**Sex**		**Asthma**	
Men	28 (35.9)	No	46 (58.7)
Women	50 (64.1)	Yes	32 (41.3)
**BMI**		**Blood Pressure**	
Normal weight	19 (24.4)	Normotensive	29 (39.7)
Overweight	30 (38.5)	Prehypertensive	23 (31.5)
Obese	29 (37.1)	Hypertensive	21 (28.8)
**Metabolic Syndrome**		**Castelli I index**	
No	47 (60.3)	<4 low risk	31 (40.3)
Yes	31 (39.7)	>4 high risk	46 (59.7)
**Type 2 diabetes**		**Castelli II index**	
No	59 (75.6)	<3 low risk	50 (64.9)
Yes	19 (24.4)	>3 high risk	27 (35.1)
**HOMA Index**		**Atherogenic coefficient**	
Without IR	27 (39.1)		
Suspected IR	17 (24.6)	<2 low risk	11 (14.3)
IR	25 (36.2)	>2 high risk	66 (85.7)
**WHtR**		**AIP**	
<0.5	13 (16.9)	<0.24 low risk	9 (11.7)
>0.5	64 (83.1)	>0.24 high risk	68 (88.3)
**Neck Circumference (cm)**		**Apo-Index**	
Normal weight	31 (41.9)	low risk	39 (65)
Overweight—Obese	43 (58.9)	high risk	21 (35)
**Waist Circumference (cm)**		**HRR1**	
Normal weight	23 (29.9)	Normal	57 (73.1)
Overweight—Obese	54 (70.1)	Abnormal	21 (26.9)
**Phase angle (°)**		**HRR2**	
>5.4	40 (51.3)	Normal	58 (74.4)
<5.4	38 (48.7)	Abnormal	20 (25.6)

Abbreviations: IQR: interquartile range; BMI: body mass index; WHtR: waist-to-height ratio; HRR1: heart rate recovery at first minute; AIP: atherogenic index of plasma.

**Table 2:** Descriptive respiratory-related measurements, bioimpedance, hematic biometry, and blood chemistry of patients recruited by National Institutes of Health, Mexico City.

**Variable**	**Total (*n* = 78)**	
	**Median (IQR)**	**Min-Max**
**Respiratory-related measurements**		
SpO2 basal (%)	95 (93–96)	87–99
SpO2 final (%)	93 (91–94)	75–98
FeNO (ppb)	15.8 (8.3–24.65)	3.33–246
**Bioimpedance**		
SMM (Kg)	18.7 (16.3–23)	10.3–33.6
Phase angle (°)	5.5 (5.0–6.0)	4.0–7.1
Intracellular water (L)	17.4 (15.5–21.9)	10.4–29.4
Hydration (%)	77 (69.9–85.1)	35.3–106.2
**Blood cell Counts**		
Leukocytes (10^3^/µL^−1^)	6.4 (5.7–7.3)	3.9–16.9
Neutrophils (10^3^/µL^−1^)	3.66 (3.2–4.3)	1.8–5.8
Lymphocytes (10^3^/µL^−1^)	1.97 (1.7–2.3)	1.3–4.0
Monocytes (10^3^/µL^−1^)	0.4 (0.4–0.5)	0.23–0.9
Eosinophils (10^3^/µL^−1^)	2.4 (1.3–3.5)	0.4–13.5
Basophils (10^3^/µL^−1^)	0.5 (0.4–1.2)	0.2–1.2
Erythrocytes (10^6^/µL^−1^)	5.18 (4.71–5.58)	4.09–6.29
Hemoglobin (gr/dL)	15.2 (14.3–16.5)	11.6–18.6
Hematocrit (%)	45.9 (42.7–49.9)	36.5–56.6
**Blood Chemistry parameters**		
Glucose (mg/dL^−1^)	97 (90–108.5)	75–289
HbA1c (%)	5.7 (5.4–6.0)	4.5–11.9
Insulin (µU/mL^−1^)	10.55 (7.6–16.2)	2.9–34.5
HOMA-IR	2.46 (1.54–3.86)	0.46–11.84
LDL (mg/dL^−1^)	120.9 (96–144)	48.7–229
No-HDL (mg/dL^−1^)	150 (124.5–180.5)	67–264
Castelli-I index	4.6 (3.5–5.3)	0.048–7.1
Castelli-II index	2.79 (2.16–3.21)	0.91–4.98
AC	3.6 (2.5–4.29)	1.2–6.1
CRP (mg/dL^−1^)	0.15 (0.07–0.42)	0.02–2.02

Abbreviations: SMM: skeletal muscle mass; SpO2: oxygen pulse saturation; FeNO: fractional exhaled nitric oxide; HOMA-IR: homeostatic model assessment insulin resistance; LDL: low-density lipoprotein; No-HDL: no high-density lipoprotein; CRP: C-reactive protein; AC: atherogenic coefficient.

**Table 3:** General characteristics of the population recruited by National Institutes of Health, Mexico City.

**Variable**	**HR (bpm)**				**HRR (bpm)**				**rHRR (%)**			
	**1 min**		**2 min**		**1 min**		**2 min**		**1 min**		**2 min**	
	**rho**	***p*** **-Value**	**rho**	***p*** **-Value**	**rho**	***p*** **-Value**	**rho**	***p*** **-Value**	**rho**	***p*** **-Value**	**rho**	***p*** **-Value**
SpO2b (%)	−0.17	0.139	−0.048	0.675	0.309	0.006	0.129	0.257	0.29	0.010	0.089	0.440
SpO2f (%)	−0.23	0.046	−0.195	0.087	0.059	0.602	0.017	0.884	0.09	0.425	0.045	0.696
FeNO (ppb)	0.001	0.990	−0.05	0.666	−0.22	0.060	−0.209	0.070	−0.17	0.145	−0.14	0.237
**Variable**	**Total**		**Biomarker**		**Normal**		**Abnormal**				***p*** **-Value**	
	**Median (IQR)**				**Median (IQR)**		**Median (IQR)**					
SpO2b (%)	95 (93–96)		HRR1		95 (94–96)		93 (92–95)				0.005	
			HRR2		95 (93–96)		93.5 (92.5–95)				0.198	
SpO2f (%)	93 (91–94)		HRR1		93 (92–95)		92 (91–94)				0.031	
			HRR2		93.5 (92–94)		92 (91–94)				0.134	
FeNO (ppb)	15.8 (8.3–24.65)		HRR1		15.5 (8.3–22.7)		18.2 (9.6–37.3)				0.267	
			HRR2		11.6 (8.3–23)		18.6 (11–43.3)				0.043	

Abbreviations: HRR, heart rate recovery; Abnormal HRR1 (aHRR) and HRR2 (aHRR) were categorized using the first quartile of all the data, ≤8, and ≤11 bpm was used as cutoff point, respectively; SpO2b: basal oxygen saturation; SpO2f: final oxygen saturation; FeNO: fractional exhaled nitric oxide. IQR, interquartile range 25–75. A Spearman’s correlation test and a Wilcoxon rank-sum test were used for the statistical analysis. Spearman’s correlation coefficient is shown as rho.

**Table 4:** Correlation between heart rate, heart rate recovery at 1 and 2 min, relative heart rate recovery, and body composition parameters. Comparative differences in body composition between normal and abnormal HRR.

**Variable**	**HR (bpm)**				**HRR (bpm)**				**rHRR (%)**			
	**1 min**		**2 min**		**1 min**		**2 min**		**1 min**		**2 min**	
	**rho**	***p*** **-Value**	**rho**	***p*** **-Value**	**rho**	***p*** **-Value**	**rho**	***p*** **-Value**	**rho**	***p*** **-Value**	**rho**	***p*** **-Value**
SMM (Kg)	0.146	0.203	0.119	0.297	−0.207	0.069	−0.143	0.212	−0.234	0.039	−0.192	0.092
Phase angle (ϕ)	0.229	0.043	0.237	0.037	−0.226	0.047	−0.216	0.057	−0.261	0.021	−0.275	0.015
Intracellular Water (L)	0.156	0.172	0.145	0.205	−0.193	0.090	−0.146	0.204	−0.227	0.045	−0.202	0.077
Hydration (%)	−0.166	0.146	−0.147	0.199	0.234	0.039	0.188	0.099	0.255	0.024	0.231	0.042
**Variable**	**Total**		**Biomarker**		**HRR**			**HRR**			***p*** **-Value**	
	**Median (IQR)**				**Median (IQR)**			**Median (IQR)**				
Phase angle (°)	5.5 (5–6)		HRR1		5.3 (5–5.9)			5.7 (5–6.4)			0.097	
			HRR2		5.3 (4.9–5.9)			5.7 (5.4–6.3)			0.060	
SMM (Kg)	18.7 (16.3–23)		HRR1		18 (16.24–22)			21.8 (17.1–28.9)			0.039	
			HRR2		18 (16–22.25)			21.5 (17.3–26.4)			0.158	
Intracellular water (L)	17.35 (15.5–21.9)		HRR1		17 (15.1–20.3)			20.3 (16.2–26)			0.038	
			HRR2		17.1 (15.1–21)			19.8 (16.4–23.9)			0.134	
Hydration (%)	77 (69.9–85.1)		HRR1		79 (71.4–87.9)			73 (64.2–82.3)			0.035	
			HRR2		79.8 (70–87.9)			73 (67.5–81.5)			0.082	

Abbreviations: HRR1 and HRR2: heart rate recovery at one and two minutes; aHRR: Abnormal HRR at one and two minutes were categorized using the first quartile of all the data and values of ≤8 and ≤11 bpm as cutoff points respectively; IQR: interquartile range 25–75; SMM: skeletal muscle mass; TEE: total energy expenditure. A Spearman’s correlation test and a Wilcoxon rank-sum test were used for the statistical analysis. Spearman’s correlation coefficient is shown as rho.

**Table 5:** Correlation between heart rate, heart rate recovery at 1 and 2 min, relative heart rate recovery and hematic biometry. Comparative differences in white blood cell counts and red blood cell parameters between normal and abnormal HRR.

**Variable**	**HR (bpm)**				**HRR (bpm)**				**rHRR (%)**			
	**1 min**		**2 min**		**1 min**		**2 min**		**1 min**		**2 min**	
	**rho**	***p*** **-Value**	**rho**	***p*** **-Value**	**rho**	***p*** **-Value**	**rho**	***p*** **-Value**	**rho**	***p*** **-Value**	**rho**	***p*** **-Value**
Leucocytes (10^3^/µL^−1^)	0.634	0.021	0.236	0.039	−0.389	0.001	−0.328	0.004	−0.373	0.001	−0.329	0.004
Neutrophils (10^3^/µL^−1^)	0.209	0.068	0.279	0.014	−0.234	0.041	−0.303	0.007	−0.255	0.025	−0.328	0.004
Lymphocytes (10^3^/µL^−1^)	0.192	0.095	0.115	0.321	−0.299	0.008	−0.198	0.085	−0.262	0.021	−0.182	0.114
Monocytes (10^3^/µL^−1^)	0.195	0.089	0.048	0.678	−0.371	0.001	−0.158	0.169	−0.32	0.005	−0.115	0.319
Erytrocytes (10^6^/µL^−1^)	0.173	0.134	0.127	0.272	−0.312	0.006	−0.209	0.068	−0.325	0.004	−0.233	0.042
Hemoglobin (gr/dL)	0.121	0.290	0.077	0.507	−0.319	0.005	−0.246	0.030	−0.309	0.006	−0.234	0.041
Hematocrit (%)	0.146	0.207	0.121	0.295	−0.293	0.009	−0.244	0.032	−0.297	0.009	−0.247	0.030
**Variable**	**Total**			**Biomarker**		**Normal**			**Abnormal**		***p*** **-Value**	
	**Median (IQR)**					**Median (IQR)**			**Median (IQR)**			
Leucocytes (10^3^/µL^−1^)	6.4 (5.7–7.3)			HRR1		6.1 (5.5–6.95)			7.3 (6.4–8.3)		0.0008	
				HRR2		6.3 (5.6–7.2)			6.85 (6.05–7.65)		0.0654	
Neutrophils (10^3^/µL^−1^)	3.7 (3.2–4.3)			HRR1		3.6 (3–4.1)			3.9 (3.5–4.8)		0.0214	
				HRR2		3.6 (3.1–4.2)			3.85 (3.3–4.6)		0.2379	
Lymphocytes (10^3^/µL^−1^)	2 (1.7–2.3)			HRR1		1.9 (1.59–2.27)			2.2 (1.8–2.6)		0.0254	
				HRR2		1.9 (1.6–2.3)			2.2 (1.85–2.4)		0.1331	
Monocytes (10^3^/µL^−1^)	0.4 (0.4–0.5)			HRR1		0.4 (0.335–0.5)			0.5 (0.4–0.7)		0.0117	
				HRR2		0.4 (0.36–0.5)			0.4 (0.4–0.55)		0.2697	
Erytrocytes (10^6^/µL^−1^)	5.16 (4.7–5.58)			HRR1		5.01 (4.64–5.49)			5.57 (5.21–5.68)		0.0239	
				HRR2		5.09 (4.67–5.57)			5.33 (5.04–5.67)		0.0955	
Hemoglobin (gr/dL)	15.2 (14.3–16.5)			HRR1		14.8 (13.7–16.25)			16 (15.2–17)		0.0199	
				HRR2		14.9 (13.9–16.2)			15.9 (14.9–17.05)		0.0833	
Hematocrit (%)	45.9 (42.5–49.9)			HRR1		44.65 (41.5–49.75)			47.9 (45.8–50.8)		0.0228	
				HRR2		44.8 (41.8–49)			48 (45.3–51.1)		0.0426	

Abbreviations: HRR: heart rate recovery; aHRR1 and aHRR2: Abnormal HRR at 1 and 2 min. aHRR1 and HRR2 were categorized using the first quartile of all the data and values of ≤8 and ≤11 as cutoff points; IQR: interquartile range 25–75. A Spearman’s correlation test and a Wilcoxon rank-sum test were used for the statistical analysis. Spearman’s correlation coefficient is shown as rho.

**Table 6:** Correlation between heart rate, heart rate recovery at 1 and 2 min, relative heart rate recovery, blood chemistry parameters and atherogenic indices. Comparative differences in HOMA between normal and abnormal HRR.

**Variable**	**HR (bpm)**				**HRR (bpm)**				**rHRR (%)**			
	**1 min**		**2 min**		**1 min**		**2 min**		**1 min**		**2 min**	
	**rho**	***p*** **-Value**	**rho**	***p*** **-Value**	**rho**	***p*** **-Value**	**rho**	***p*** **-Value**	**rho**	***p*** **-Value**	**rho**	***p*** **-Value**
Insulin	0.316	0.008	0.264	0.029	−0.233	0.054	−0.105	0.380	−0.248	0.040	−0.154	0.210
HOMA-IR	0.350	0.003	0.299	0.013	−0.247	0.041	−0.105	0.390	−0.268	0.026	−0.166	0.172
LDL	0.266	0.020	0.183	0.111	−0.144	0.211	−0.046	0.689	−0.200	0.082	−0.089	0.440
No-HDL	0.225	0.049	0.157	0.173	−0.128	0.267	−0.066	0.567	−0.175	0.128	−0.097	0.404
Castelli-1	0.259	0.023	0.260	0.023	−0.164	0.155	−0.188	0.101	−0.209	0.069	−0.241	0.035
Castelli-2	0.230	0.044	0.210	0.072	−0.132	0.251	−0.118	0.307	−0.170	0.139	−0.160	0.167
AC	0.242	0.034	0.250	0.028	−0.129	0.262	−0.171	0.137	−0.179	0.119	−0.222	0.052
CRP	0.332	0.004	0.239	0.042	−0.099	0.403	−0.023	0.847	−0.139	0.242	−0.077	0.517
**Variable**	**Total**		**biomarker**		**Normal**		**Abnormal**		***p*** **-Value**			
	**Median (IQR)**				**Median (IQR)**		**Median (IRQ)**					
HOMA-IR	2.46 (1.54–3.85)		HRR1		2.35 (1.31–3.6)		3.14 (1.95–4.98)		0.052			
			HRR2		2.37 (1.47–3.85)		2.8 (1.68–4.32)		0.383			

Abbreviations: HOMA-IR: homeostatic model assessment insulin resistance; LDL: low-density lipoprotein; No-HDL: no high-density lipoprotein; CRP: C-reactive protein; AC: atherogenic coefficient; HRR: heart rate recovery. Abnormal HRR1 (aHRR) and HRR2 (aHRR) were categorized using the first quartile of all the data, 8, and 11 bpm as cutoff points, respectively; IQR, interquartile range 25–75. A Spearman’s correlation test and a Wilcoxon rank-sum test were used for the statistical analysis.

## Data Availability

The data used to support the findings of this study are available from the core.
